# Hierarchical Assembly of SnO_2_/ZnO Nanostructures for Enhanced Photocatalytic Performance

**DOI:** 10.1038/srep11609

**Published:** 2015-06-25

**Authors:** Liangliang Zhu, Minghui Hong, Ghim Wei Ho

**Affiliations:** 1 Department of Electrical and Computer Engineering, National University of Singapore, 4 Engineering Drive 3, 117583, Singapore; 2 Engineering Science Programme, National University of Singapore, 9 Engineering Drive 1, 117575, Singapore; 3 Institute of Materials Research and Engineering, A*STAR (Agency for Science, Technology and Research), 3 Research Link, 117602, Singapore

## Abstract

SnO_2_/ZnO hierarchical heterostructures have been successfully synthesized by combining electrospinning technique and hydrothermal method. Various morphologies of the secondary ZnO nanostructures including nanorods (NRs) and nanosheets (NSs) can be tailored by adding surfactants. Photocatalytic performance of the heterostructures was investigated and obvious enhancement was demonstrated in degradation of the organic pollutant, compared to the primary SnO_2_-based nanofibers (NFs) and bare ZnO. Furthermore, it was found that the H_2_ evolution from water splitting was achieved by photocatalysis of heterostructured nanocomposites after sulfurization treatment. This synthetic methodology described herein promises to be an effective approach for fabricating variety of nanostructures for enhanced catalytic applications. The heterostructured nanomaterials have considerable potential to address the environmental and energy issues *via* degradation of pollutant and generation of clean H_2_ fuel.

The global issues of environmental problem and energy shortage have affected human live and health^1^,^2^. Photocatalysis as an efficient and green approach has shown great potential in resolving aforementioned issues using the inexhaustible solar energy^3^. In recent years, many kinds of semiconductor metal oxide photocatalysts, such as TiO_2_^4^, SnO_2_^5^, ZnO^6^, In_2_O_3_^7^ nanocatalysts, and so forth, have been developed. However, enhancing the photocatalytic efficiency of those semiconductors to meet the practical application requirements is still a challenge because of poor light absorption and the rapid recombination of photogenerated electrons and holes caused by the unsuitable band gap of individual materials^8^. The hierarchical assembly of nanoscale building photocatalyst blocks with a tunable dimension and structure complexity offers an essential strategy toward the realization of multifunctionality of nanomaterials in order to enhance the photocatalytic activity by extending the photoresponse range and increase the electron-hole pair separation efficiency^9^,^10^. Generally, hierarchical heterostructures are assembled from two different materials with low-dimensional nanostructures, such as nanoparticles, nanorods, nanowires, nanotubes, nanobelts, and nanosheets, providing an ultrahigh specific surface area and a network system consisting of parallel connective paths and interconnections of dissimilar functional components^11^. Therefore, a large number of semiconductor heterostructural nanomaterials, such as TiO_2_-SnO_2_^12^, CdS-ZnO^9^, ZnS-ZnO^10^, Te-SnO_2_^11^ etc., have been investigated and used for many photocatalytic reactions. Among the oxide semiconductors, ZnO and SnO_2_ are well-known wide direct band gap (Eg = 3.37 and 3.6 eV at 300 K, respectively) semiconductors and possess wide application potentials in photovoltaics, photocatalysis and gas sensing^5^,^8^,^12^. It has been demonstrated that the combination of the ZnO and SnO_2_ could hinder the charge recombination and improve the photocatalytic efficiency due to their special band gap position^8^.

On the other hand, one dimensional (1D) nanomaterials are ideal material to serve as the backbone in hierarchical heterostructures due to the enhanced charge carrier mobility and large surface area^13^,^14^,^15^. Electrospinning technique used to fabricate continuous 1D nanofibers (NFs) has shown its high efficiency and convenience^16^. Considerable attempts have been made to synthesize a variety of continuous semiconductor metal oxide NFs with the flexible diameter control from nanometers to a few micrometers^17^,^18^,^19^. These works imply that it is possible to fabricate the semiconductor-semiconductor hierarchical heterostructures with enhanced photocatalytic activity from NF backbones.

In this work, we present a facile strategy for fabricating SnO_2_/ZnO hierarchical nanostructures for enhanced photocatalytic performance, the overall procedure was shown in Fig. 1. First, the SnO_2_-based NFs used as backbone in hierarchical heterostructure were prepared by electrospinning method. Subsequently, after dip-coating with ZnO seed nanoparticles, ZnO nanorod (NR) and nanosheet (NS) secondary structures were assembled directly to the backbones of nanofibers by hydrothermal process. It is found that the SnO_2_/ZnO heterostructural nanocomposites exhibit excellent photocatalytic activity superior to the individual SnO_2_ and ZnO *via* the photocatalytic degradation of dye wastewater, which could be ascribed to the formation of heterojunction of SnO_2_-ZnO. Finally, after sulfurization treatment, these sulfurized heterostructured nanocomposites were extended to apply to solar hydrogen (H_2_) evolution, manifesting the favorable capacity and stability for photocatalytic reactions. Our demonstration of rational fabrication of hierarchical nanostructures may provide the possibility for design and preparation of photocatalysts with tunable morphology and improved photocatalytic properties applied in environmental and energy sectors.

## Results and discussion

**Figure 2:**
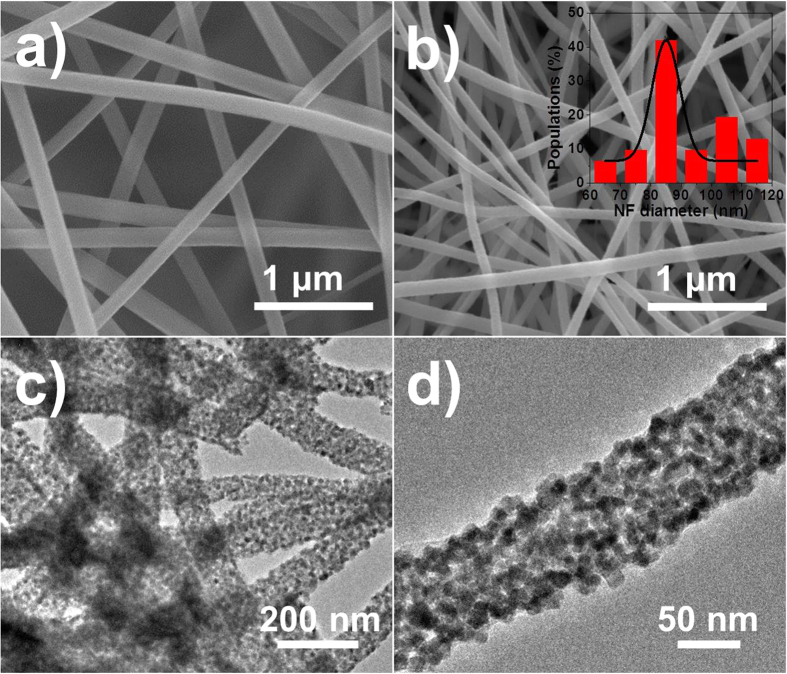
Morphology of SZ-10 NFs. The SEM images of (**a**) before and (**b**) after anneal. The inset of panel (**b**) depicts the NFs diameter histogram. Representative TEM images of SZ-10 NFs at (**c**) low and (**d**) high resolution.

Figure 2a and b show the SEM images of the as-spun SnO_2_/ZnO NF composite which is referred to as SZ-10 before and after annealing. The Sn-Zn-PVP NFs with average diameter of *ca.* 150 nm interweave randomly, constructing a three-dimensional (3D) porous mat. After annealing, PVP was removed and at the same time Sn-Zn precursors were converted to the SnO_2_/ZnO NFs. Like the pure SnO_2_ NFs (Supplementary Fig. S1), the fibrous structure of SZ-10 NFs is well preserved with an average diameter exhibited shrinkage to *ca.* 90 nm (Fig. 2b inset) resulting from the decomposition of PVP. The typical TEM images of SZ-10 NFs were illustrated in Fig. 2c,d. It could be seen that the SZ-10 NFs were composed of interconnected nanoparticles. In Fig. 2d, many nanopores were observed within the spaces between adjacent nanoparticles.

**Figure 3:**
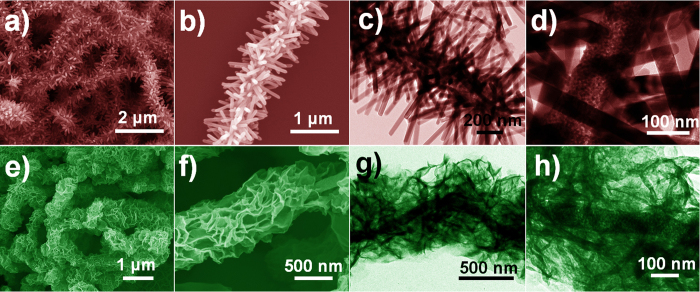
Morphologies of the hierarchical nanostructures. The SEM and TEM images of (a, b, c and d) SZ-10/ZnO NRs and (e, f, g and h) SZ-10/ZnO NSs.

After the secondary growth of ZnO NRs, the initially pure SnO_2_ (Supplementary Fig. S2) and SZ-10 NFs (Fig. 3a,b) are evenly branched out, forming hierarchical structures. The ZnO NRs with the length of *ca.* 450 nm and diameter of *ca.* 50 nm stand uniformly and densely on surfaces of the SZ-10 NFs after 2 h hydrothermal treatment (Fig. 3a,b). However, bare ZnO NR clusters (Supplementary Fig. S2a) as well as sparsely distributed ZnO NRs on the SnO_2_ NFs (Supplementary Fig. S2b) are observed, which may attribute to the less ZnO seed nanoparticles comparing to SZ-10 NFs. Furthermore, in addition of sodium citrate in precursor solution of SZ-10 NFs case, ZnO NSs generated instead of ZnO NRs. As shown in Fig. 3e,f, core-shell structure with a diameter of 800 to 1000 nm can be clearly observed, which is significantly larger than that of the plain SZ-10 NFs backbone. According to the calculation, the thickness of the sheath is around 400 nm. The corresponding TEM images reveal the detailed morphology and structure of SZ-10/ZnO NRs (Fig. 3c,d) and SZ-10/ZnO NSs (Fig. 3g,h). Both in SZ-10/ZnO NRs and SZ-10/ZnO NSs cases, SZ-10 NFs is the structure core, while secondary ZnO NRs fully covered and firmly anchored on the SZ-10 NFs, which suggests high structural stability. In addition, the high resolution TEM image of the junction between SZ-10 NFs and ZnO NR branch was taken and shown in the Supplementary Fig. S3. Both the branch and backbone display the clear lattice fringes. The measured lattice distances of 0.319 nm correspond to the SnO_2_ backbones and 0.512 nm belong to the secondary ZnO NRs^12^,^20^,^21^. Besides that, the lattice spacing of 0.325 nm in the backbone attribute to ZnO which consist in the SZ-10 NFs^12^,^20^.

**Figure 4:**
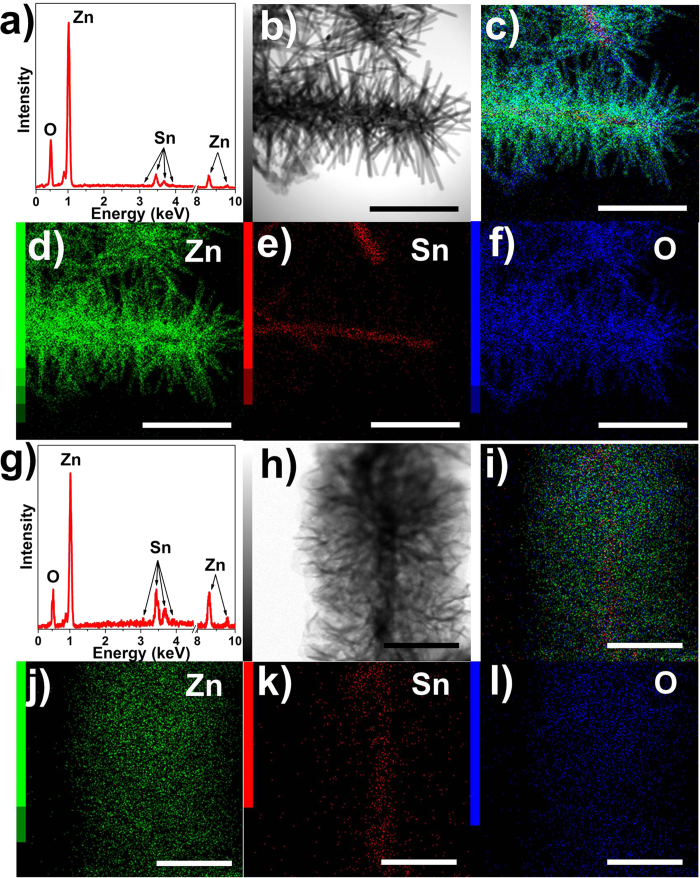
Elemental analysis of the hierarchical nanostructures. (**a**) EDX spectrum and elemental mapping images of SZ-10/ZnO NRs for the area of (**b**) for (**c**) overlay, (**d**) Zn element, (**e**) Sn element and (**f**) O element (The scale bar in (**b**-**f**) is 1 μm). (g) EDX spectrum and elemental mapping images of SZ-10/ZnO NSs for the area of (**h**) for (**i**) overlay, (**j**) Zn element, (**k**) Sn element and (**l**) O element (The scale bar in (**h**-**l**) is 500 nm).

The EDX spectra in Fig. 4a,g show that the Sn L1 peak at 3.06 keV, Lα peak at 3.44 keV, Lβ peak at 3.68 keV and Lβ_2_ peak at 3.90 keV, as well as O Kα peak at 0.52 keV are uniform both in SZ-10/ZnO NRs and NSs, which reveal that the ZnO growth process does not affect the SnO_2_ structures or properties^22^,^23^. Zn Lα_1,2_ peak at 1.01 keV, Kα_1_ peak at 8.63 keV and Kβ_1_ peak at 9.57 keV were observed indicating the successful synthesis of ZnO^9^,^12^. Elemental mapping images presented in Figs 4b–f and h–l demonstrate the well-defined spatial distribution of Zn (Fig. 4d,j), Sn (Fig. 4e,k) and O (Fig. 4f,l). The signal of Sn element is seen mainly coming from the backbone portion, while the signals of Zn and O are uniformly distributed throughout the entire heterostructure, confirming the ZnO NRs branches and NSs homogeneously covered on the SZ-10 NFs backbone.

**Figure 5:**
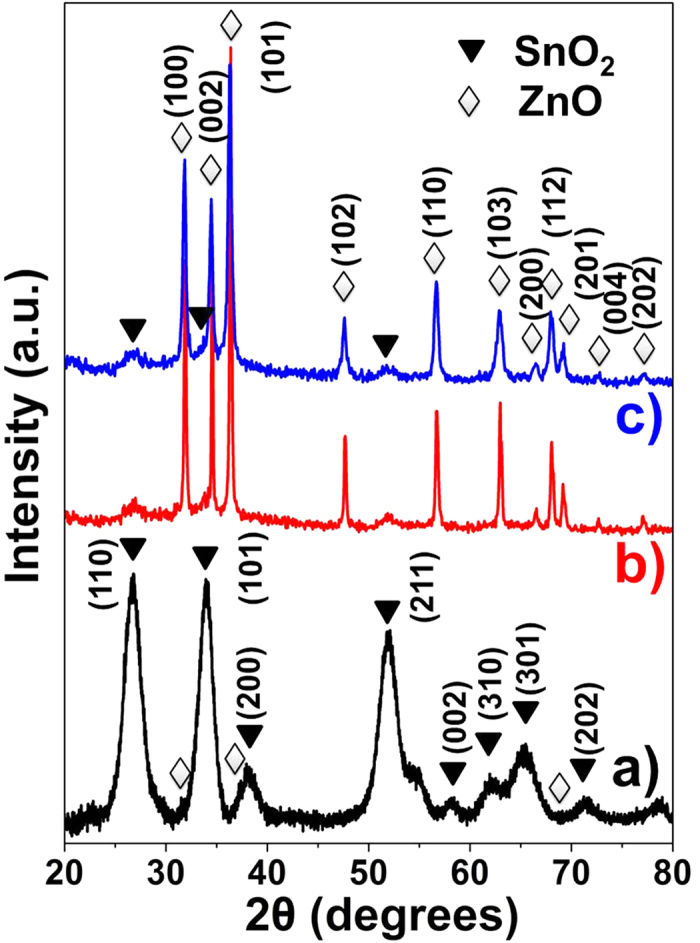
The XRD spectra of (a) SZ-10 NFs, (b) SZ-10/ZnO NRs and (c) SZ-10/ZnO NSs.

The crystal structures of SZ-10 NFs, SZ-10/ZnO NRs and SZ-10/ZnO NSs composites were also investigated by XRD and the patterns are shown in Fig. 5. The diffraction peaks in Fig. 5a at 26.6, 33.9, 38.1, 51.8, 58.0, 61.9, 66.1 and 71.5° can be indexed to the (110), (101), (200), (211), (002), (310), (301) and (202) crystal planes of the rutile phase SnO_2_ (JCPDS No. 41-1445). Meanwhile, compared to XRD spectrum of pure SnO_2_ (Supplementary Fig. S4), the SZ-10 NFs spectrum shows weak ZnO peaks at 31.5, 36.7 and 69.1° which indicates the incorporation of ZnO into the SnO_2_ NFs. After secondary growth of ZnO nanostructures, strong crystalline peaks observed at 31.9, 34.5, 36.4, 47.6, 56.7, 62.9, 66.5, 68.0, 69.2, 72.7 and 77.1^o^ correspond to the (100), (002), (101), (102), (110), (103), (200), (112), (201), (004) and (202) crystal planes of the wurtzite ZnO structure (JCPDS No. 36-1451). Weak SnO_2_ peaks were also observed at 26.6, 33.9 and 51.8°, suggesting that the composition with hierarchical structure is SnO_2_ and ZnO, as shown in Fig. 5b,c.

The corresponding UV-vis absorption measurements of SZ-10/ZnO NRs and NSs were carried out and the spectra are shown in Supplementary Fig. S5. In general, the absorbance intensities increase after the growth of ZnO hierarchical structures while the SZ-10 NFs exhibits the lowest intensity. The absorbance of SZ-10/ZnO NRs is higher than that of SZ-10/ZnO NSs, which may lead to better photocatalytic performance.

The growth process of ZnO NR branch was investigated by changing the hydrothermal treatment time. Supplementary Fig. S6 depicts the SEM images of hierarchical SZ-10/ZnO NRs nanostructures by increasing the hydrothermal time (0.5, 1, 1.5, 2 and 2.5 h). At the beginning, short and sparse ZnO NRs were grown from the nucleation sites of ZnO seed nanoparticles that were dip coated on the backbone of the nanofibers (Supplementary Fig. S6a). Over time, the absorption of Zn^2+^ and OH^−^ from the feedstock solution increases the length and density of secondary ZnO NRs (Supplementary Fig. S6b-e). However, when the reaction time was prolonged to 2.5 h, bare ZnO nanoclusters formed, as shown in Supplementary Fig. S6e inset, which are adverse to photocatalytic performance. By further measuring the diameter and length of the secondary ZnO NRs, we found that the diameter of the ZnO NRs increase slowly, from *ca.* 40 nm to *ca.* 53 nm, while the length grows dramatically, increasing from *ca.* 130 nm to *ca.* 500 nm (Supplementary Fig. S6f). This formation mechanism could be explained by the general route to grow vertical ZnO NRs array on substrates using ZnO seeds. The SZ-10 NF backbones were dip coated with a layer of ZnO seed nanoparticles to form the nucleation sites, from where the randomly oriented crystals grew. In the absence of structure modifiers, the ZnO NRs nucleate and crystallize along the [001] direction which is the fastest growth orientation on these ZnO sites. Therefore, the branched nanostructure was obtained^9^,^12^.

Citrate anions as the structure modifying agent was applied to promote the alteration of the secondary ZnO nanostructure because it adsorbs strongly on metal and mineral surfaces and significantly changes the surface properties and mineral growth behavior^24^,^25^,^26^. Sodium citrate was used in our experiments, the molecules of which absorb preferentially on the [001] surfaces and thus inhibit the crystal growth along [001] orientations^12^,^20^. As a result, ZnO NSs were formed on the surface of the SZ-10 NFs backbones. Different concentrations of sodium citrate (0.3, 0.1, 0.05 and 0.025 mol/L) were added and the corresponding SEM images (Supplementary Fig. S7) indicate the optimum sodium citrate concentration is 0.05 mol/L.

**Figure 6:**
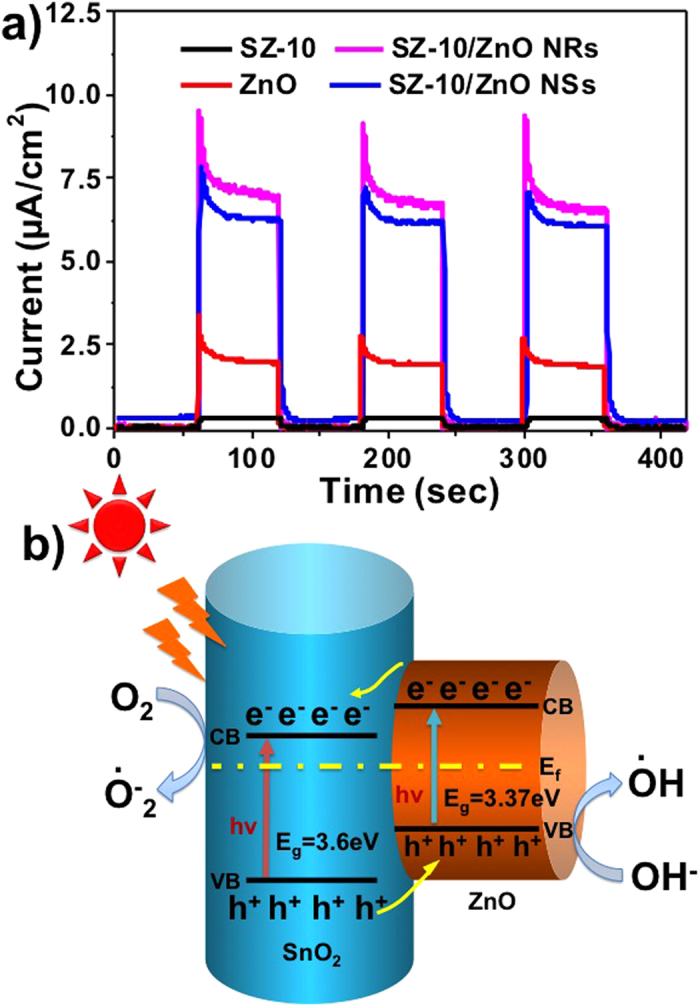
(**a**) Photocurrent responses of the PEC cells using SZ-10, bare ZnO, SZ-10/ZnO NRs and SZ-10/ZnO NSs composites as working electrodes for three 60 s light-on–off cycles. (**b**) The energy band structure diagram of the SnO_2_-ZnO.

To explore the photocurrents of the composites with hierarchical nanostructures, the PEC cells of SZ-10 NFs, bare ZnO and SZ-NFs/ZnO nanocomposites were employed under simulated sunlight glowed from Xe arc lamp and their amperometric *I–t* curves acquired with three light on/off cycles within 60 s are shown in Fig. 6. The photocurrents of both SZ-10 NFs and bare ZnO are relatively lower. The photocurrent of SZ-10 NFs is about 0.5 μA cm^−2^, while that of the SZ-10 NFs peaked at *ca.* 3.6 μA cm^−2^ and then decayed to a steady state of *ca.* 2.3 μA cm^−2^ after 30 s. However, when ZnO nanostructures are hierarchically assembled with SZ-10 NFs, the photocurrents are obviously improved comparing to that of individual NRs and NSs nanostructures. The initial photocurrents reached *ca.* 10 and 7.6 μA cm^−2^ and eventually approached to a plateau at *ca.* 7.5 and 6.5 μA cm^−2^ for SZ-10/ZnO NRs and SZ-10/ZnO NSs, respectively. This improvement in the photocurrent output is mainly due to the heterojunction formation which promotes efficient separation of photo excited electron-hole pairs and thus enhances the photocatalytic stability^27^,^28^,^29^.Figure. 6b elucidates the energy band diagram of the SnO_2_-ZnO heterojunction schematically where a type-II heterostructure with a staggered alignment is formed for the hierarchical SnO_2_/ZnO nanofibers. Under light illumination, the generation of electron–hole pairs occurs and the photogenerated electrons are transferred from the CB of ZnO to the CB of SnO_2_ and, conversely, the photogenerated hole transfer takes place from the VB of SnO_2_ to the VB of ZnO. The internal field at the SnO_2_/ZnO interface facilitates the formation of charge transfer state and the spatial separation of the photogenerated carriers within the nanofibers^5^,^8^,^30^,^31^.

**Figure 7:**
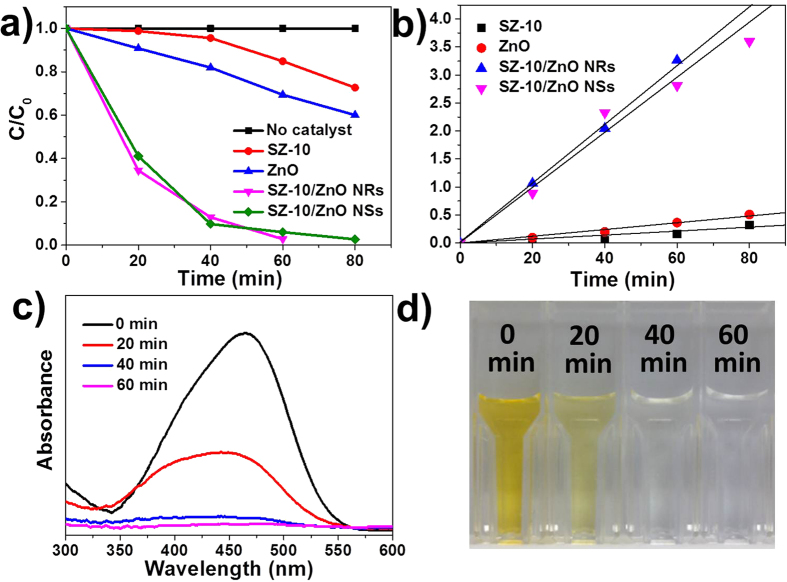
Degradation of MO solution. (**a**) Degradation kinetics and (**b**) pseudo-first order kinetics of time evolution MO photodegradation study in absence and presence of various photocatalysts. (**c**) The UV-Vis spectra and (**d**) corresponding digital photographs illustrating time MO photodegradation using SZ-10/ZnO NRs.

The photocatalytic performance was demonstrated by photocatalytic degradation of MO, a common textile pollutant^32^,^33^. Figure 7a exhibits the content changes of MO in the absence and in the presence of different composites. A control experiment was carried out to show that photodegradation is not apparent in the absence of the photocatalyst. Also, photocatalytic degradation capabilities of SZ-10 NFs backbone and bare ZnO were tested and the results show 27.4% and 39.8% of MO molecules were degraded after 80 min of simulated sunlight irradiation respectively. In contrast, both SZ-10/ZnO NRs and SZ-10/ZnO NSs composites show appreciable photocatalytic activity under simulated sunlight and were able to fully degrade the MO dye after 60 and 80 min, respectively. The UV-Vis absorbance and digital photograph illustrating the time evolution photodegradation study using SZ-10/ZnO NRs are shown in Fig. 7c,d, which is clearly observed the MO solution recovered to clear after 60 min. Figure 7b shows the pseudo-first order kinetics of the MO degradation of the various photocatalysts. The efficiency of MO photodegradation by the composite was determined quantitatively using the pseudo-first order model^34^ as follows:


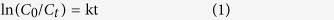


where C_0_ and C_t_ are the concentrations of dye at time 0 and t, respectively and k is the pseudo-first order rate constant. The constants k of SZ-10 NFs backbone and bare ZnO are 0.0034 and 0.0059 min^−1^, while the constants k of SZ-10/ZnO NRs and SZ-10/ZnO NSs composite photocatalysts are 0.0528 and 0.0493 min^−1^, respectively, demonstrating the hierarchical composites exhibit enhanced photodegradation. The degradation of MO might be attributed to the production of superoxide radical anion (O_2_^−^•) because the electrons in the conduction band on the photocatalyst surface reduce oxygen molecules to the superoxide anion. The active hydroxyl radicals (HO•) are generated by the reaction of O_2_^−^• and holes under irradiation. These radicals were generally assumed to be the main oxidizing agents promoting the photooxidation of MO molecules on the surface of photocatalyst^35^,^36^.

**Figure 8:**
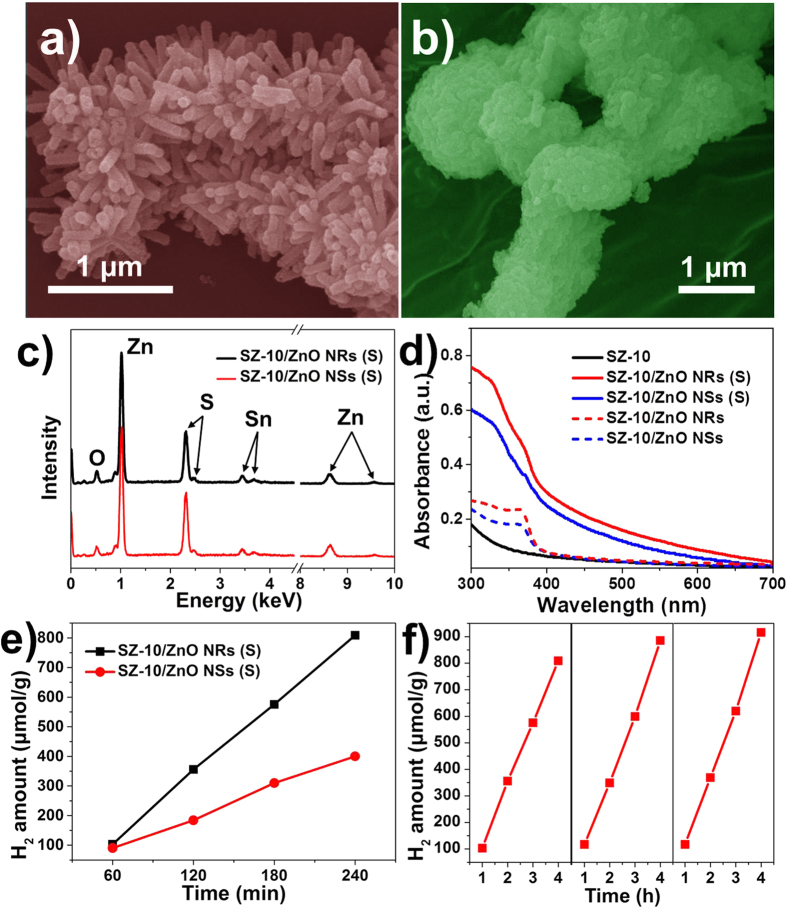
Morphology and photocatalytic performance of sulfurized hierarchical nanostructures. The SEM images of (**a**) SZ-10/ZnO NRs (S) and (**b**) SZ-10/ZnO NSs (S). (**c**) EDX spectra and (**d**) UV-vis absorbance spectra of SZ-10/ZnO NRs (S) and SZ-10/ZnO NSs (S). (**e**) H_2_ amount over the irradiation time for SZ-10/ZnO NRs (S) and SZ-10/ZnO NSs (S). (**f**) A typical time course of H_2_ production from SZ-10/ZnO NRs (S) for 3 cycles.

Finally, in order to extend the application of photocatalysts to energy field, namely the solar hydrogen production, both SZ-10/ZnO NR and SZ-10/ZnO NS nanocomposites were sulfurized using Na_2_S solution, referred to as SZ-10/ZnO NRs (S) and SZ-10/ZnO NSs (S) respectively. Figure 8a,b show the SEM images of SZ-10/ZnO NRs (S) and SZ-10/ZnO NSs (S). It is found that SZ-10/ZnO NRs (S) can retain the overall morphology except that the secondary ZnO NRs structures are shorter in length and have rougher surface (Fig. 8a). However, SZ-10/ZnO NSs (S) loss their original morphology as the NSs collapsed and transformed into agglomerated nanoparticles sheath. (Fig. 8b) The elemental EDX examinations were carried out and corresponding spectra are shown in the Fig. 8c. Compared to the EDX spectra of SZ-10/ZnO NRs and SZ-10/ZnO NSs, additional sulfur elements appear in both spectra of SZ-10/ZnO NRs (S) and SZ-10/ZnO NSs (S), meanwhile, the signal intensity of oxygen element decreased relatively, which prove the sulfurization of the nanostructures. Figure 8d depicts the UV-vis absorbance spectra of SZ-10/ZnO NRs (S) and SZ-10/ZnO NSs (S). Compared to pristine samples, the absorbance of both sulfurized samples have broadened the absorption spectra and extended to the visible-light region^10^. Moreover, the absorbance property of SZ-10/ZnO NRs (S) is higher than that of SZ-10/ZnO NSs (S). The results indicate that the sulfurized samples may show the enhanced photocatalytic activity in solar hydrogen generation and SZ-10/ZnO NRs (S) has a higher performance.

The H_2_ evolution from water splitting was investigated under simulated sunlight by utilizing these nanocomposites as photocatalysts^37^,^38^. Figure 8e illustrates the time course of H_2_ production of the SZ-10/ZnO NRs (S) and SZ-10/ZnO NSs (S). The SZ-10/ZnO NRs (S) shows a higher H_2_ evolution rate of 202.2 μmol g^−1^ h^−1^ than that of SZ-10/ZnO NSs (S) at the rate of 100.1 μmol g^−1^ h^−1^, which is in accordance with the UV-vis absorbance. The reusability of the photocatalyst for sacrificial water splitting by SZ-10/ZnO NRs (S) was demonstrated (Fig. 8f) hence suggesting that the structural stability of nanocomposite for promising solar hydrogen generation application.

## Conclusion

In summary, SnO_2_/ZnO hierarchical nanostructures have been successfully fabricated by combining electrospinning technique and hydrothermal method. The NR and NS morphologies of secondary ZnO nanostructure can be tuned by adjusting the reaction time and modifying agents. The as-prepared SZ-10/ZnO NR and NS composites have been explored for degradation of organic pollutant in water. Both NR and NS composites show much higher efficiency than primary SZ-10 NFs and bare ZnO, indicating the enhanced photocatalytic performance. More interestingly, the heterostructures can be extended to H_2_ production from sacrificial water splitting after sulfurization treatment and show favorable reusability and structural stability. Therefore, such approach towards the growth of hierarchical nanostructures may offer an avenue for designing and fabricating other complicated nanostructures and nanocomposites, which might be useful for photocatalytic application in environment and energy field.

## Methods

### Materials

Tin (II) chloride dihydrate (SnCl_2_·2H_2_O), zinc nitrate hexahydrate (Zn(NO_3_)_2_·6H_2_O), polyvinylpyrrolidone (PVP; M_w_ = 1,300,000), hexamethylenetetramine (HMT), sodium citrate, methyl orange (MO), sodium sulfate (Na_2_SO_4_), sodium sulfite (Na_2_SO_3_), sodium sulfide (Na_2_S), N,N-Dimethylformamide (DMF) and ethanol were purchased from standard sources. All chemicals were used as received without further purification.

### Fabrication of SnO_2_-based NFs

SnO_2_-based composite NFs were prepared by the electrospinning technique^8^,^30^,^39^. Typically, 0.9 g SnCl_2_·2H_2_O and 0.09 g Zn(NO_3_)_2_·6H_2_O were added into the 5 g of ethanol and 6 g DMF solution under vigorously stirring for 3 h at 60 °C. Then, 1.2 g of PVP was added and the mixture was subsequently magnetic stirred for more 3 h at the same temperature to obtain a homogenous and clear Sn-Zn-PVP precursor solution. Subsequently, electrospinning was carried out at an applied voltage of 18 kV and a flow rate of 1 mL h^−1^. The distance between needle tip and aluminum foil collector was 15 cm. Finally, the as-spun NFs were calcined in air at 500 °C for 2 h in a furnace, at temperature ramp rate of 5 °C min^−1^ to obtain SnO_2_/ZnO NFs.

### Synthesis of the SZ-10/ZnO hierarchical structure

SZ-10/ZnO NRs were prepared by a hydrothermal process^12^. First, the SZ-10 NFs were coated with ZnO nanoparticles which were prepared according to a previous work^40^ as seeds by a dip-coating technique. Subsequently, the SZ-10 NFs were dispersed into a 30 mL aqueous solution of equimolar Zn(NO_3_)_2_·6H_2_O and HMT. The hydrothermal treatment was conducted at 90 °C for 2 h. After reactions, the products were separated and washed with deionized water by centrifugation for 3 times. The resulting precipitates were dried in the drying oven. SZ-10/ZnO NSs were also synthesized as described above by adding sodium citrate as the surfactant into the hydrothermal solution and heated for 6 h.

### Characterization of hierarchical structure

The fabricated products were analyzed using an X-ray diffractometer (XRD, Philips X-ray diffractometer with Cu Kα radiation at λ = 1.541 Å) to obtain crystallographic information. Morphology and structural characteristics were studied using a field-emission scanning electron microscope (FESEM, JEOL FEG JSM 7001F) and a transmission electron microscope (TEM, Phillips FEG CM300), respectively. The elements were analyzed by energy-dispersive X-ray spectroscopy (EDX, Oxford Instruments). The absorption spectra of photocatalysts were obtained using a UV-visible spectrophotometer (UV-vis, Shimadzu UV-3600).

### Photocurrent test of photoelectrochemical (PEC) cells

The PECs were realized by coating a layer of bare ZnO, SZ-10 NFs, SZ-10/ZnO NR and NS nanocomposites on fluorine doped tin oxide (FTO) glasses (1.5 × 2 cm) and calcined for 10 min on a 450 °C hotplate. With the 0.1 mol/L Na_2_SO_4_ solution as electrolyte, the sample as working electrode and Pt as the reference electrode, the amperometric curves *I-t* were recorded under an illumination with the simulated sunlight glowed from 300 W Xe arc lamp of intensity 100 mW cm^−2^ for three 60 s light-on-off cycles.

### Degradation of MO solution

The photocatalytic reactions of 15 ml of 10 mg L^−1^ MO aqueous solution was carried out based on 15 mg of under light irradiation with the same Xe arc lamp. The concentration of MO was analyzed using a UV-Vis spectrophotometer and the maximal absorbance peak value was noted to plot the amount of MO degraded and thus, determine the photodegradation activity of the composite.

### Sulfurization of SZ-10/ZnO heterostructures

In order to prepare sulfurized SZ-10/ZnO heterostructures, the as-prepared SZ-10/ZnO NR and NS nanocomposites (20 mg) were dispersed in 10 mL of 1M Na_2_S solution and sonicated for 10 min. The mixtures were then sealed in a glass bottle before being heated at 70 °C for 4 h. After the reaction, the products were collected by the rinse–centrifugation process with deionized water for several times. The obtained products were dried thoroughly at 55 °C in oven for 4 h^41^.

### Photocatalytic water splitting

The photocatalytic reactions were conducted in a quartz cylindrical reaction cell of 25 mL in volume. Typically, 5 mg of photocatalyst, 9 mL deionized water and 1 mL methanol were mixed and stirred for 30 min to form a homogeneous suspension. The reactor was purged with argon (Ar) gas for 10 min prior to illumination with the same Xe arc lamp. Gas samples were analysed using a gas chromatograph (Shimadzu GC-2014AT).

## Additional Information

**How to cite this article**: Zhu, L. *et al.* Hierarchical Assembly of SnO_2_/ZnO Nanostructures for Enhanced Photocatalytic Performance. *Sci. Rep.* **5**, 11609; doi: 10.1038/srep11609 (2015).

## Supplementary Material

Supplementary Information

## Figures and Tables

**Figure 1:**
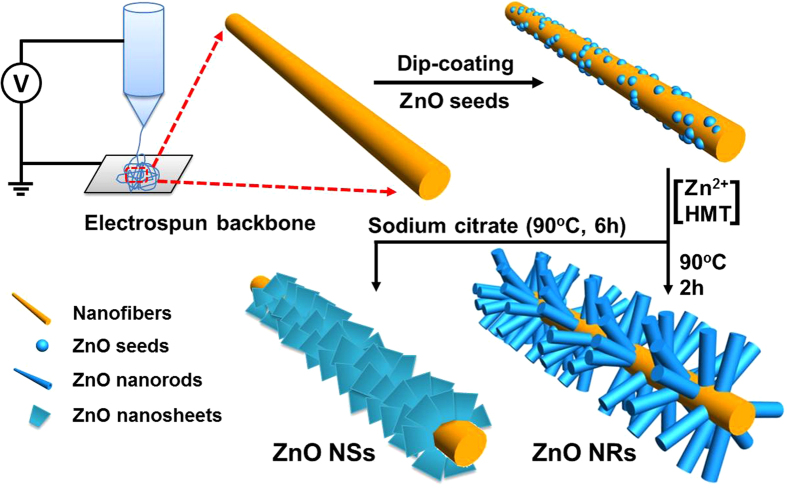
Schematic illustrations for assembly of SnO_2_/ZnO hierarchical nanostructures.

## References

[b1] Kudo A. &Miseki Y. Heterogeneous photocatalyst materials for water splitting. Chem.Soc.Rev. 38, 253–278 (2009). 19088977 10.1039/b800489g

[b2] Walter M. G. *et al.* Solar Water Splitting Cells. Chem. Rev. 110, 6446–6473 (2010). 21062097 10.1021/cr1002326

[b3] Li Z. S., Luo W. J., Zhang M. L., Feng J. Y. & Zou Z. G. Photoelectrochemical cells for solar hydrogen production: current state of promising photoelectrodes, methods to improve their properties, and outlook. Energy Environ. Sci. 6, 347–370 (2013).

[b4] Kawai T. & Sakata T. Hydrogen evolution from water using solid carbon and light energy. Nature 282, 283–284 (1979).

[b5] Lee J. S., Kwon O. S. & Jang J. Facile synthesis of SnO_2_ nanofibers decorated with N-doped ZnO nanonodules for visible light photocatalysts using single-nozzle co-electrospinning. J. Mater. Chem. 22, 14565–14572 (2012).

[b6] Ong W. L. *et al.* Highly flexible solution processable heterostructured zinc oxide nanowires mesh for environmental clean-up applications. RSC Adv. 4, 27481–27487 (2014).

[b7] Wang D. F., Zou Z. G. & Ye J. H. Photocatalytic Water Splitting with the Cr-Doped Ba_2_In_2_O_5_/In_2_O_3_ Composite Oxide Semiconductors. Chem. Mater. 17, 3255–3261 (2005).

[b8] Zhang Z. Y. *et al.* Electrospun Nanofibers of ZnO-SnO_2_ Heterojunction with High Photocatalytic Activity. J. Phys. Chem. C 114, 7920–7925 (2010).

[b9] Zhang C. *et al.* Photosensing performance of branched CdS/ZnO heterostructures as revealed by *in situ* TEM and photodetector tests. Nanoscale 6, 8084–8090 (2014). 24915978 10.1039/c4nr00963k

[b10] Tian W. *et al.* Flexible Ultraviolet Photodetectors with Broad Photoresponse Based on Branched ZnS-ZnO Heterostructure Nanofilms. Adv. Mater. 26, 3088–3093 (2014). 24523228 10.1002/adma.201305457

[b11] Her Y. C., Yeh B. Y. & Huang S. L. Vapor−Solid Growth of p-Te/n-SnO_2_ Hierarchical Heterostructures and Their Enhanced Room-Temperature Gas Sensing Properties. ACS Appl. Mater. Interfaces 6, 9150–9159 (2014). 24848993 10.1021/am5012518

[b12] Cheng C. W. *et al.* Hierarchical Assembly of ZnO Nanostructures on SnO_2_ Backbone Nanowires: Low-Temperature Hydrothermal Preparation and Optical Properties. ACS nano 3, 3069–3076 (2009). 19772329 10.1021/nn900848x

[b13] Wu H. *et al.* Electrospun Metal Nanofiber Webs as High-Performance Transparent Electrode. Nano Lett. 10, 4242–4248 (2010). 20738115 10.1021/nl102725k

[b14] Wu H. *et al.* A transparent electrode based on a metal nanotrough network. Nat. Nanotechnol. 8, 421–425 (2013). 23685985 10.1038/nnano.2013.84

[b15] Le V. T. *et al.* Coaxial Fiber Supercapacitor Using All-Carbon Material Electrodes. ACS nano 7, 5940–5947 (2013). 23731060 10.1021/nn4016345

[b16] Zhu L. L., Hong M. H. & Ho G. W. Fabrication of wheat grain textured TiO_2_/CuO composite nanofibers for enhanced solar H_2_ generation and degradation performance. Nano Energy 11, 28–37 (2015).

[b17] Hou H. L. *et al.* General Strategy for Fabricating Thoroughly Mesoporous Nanofibers. J. Am. Chem. Soc. 136, 16716–16719 (2014). 25407313 10.1021/ja508840c

[b18] Xu S. Y., Poirier G. & Yao N. Fabrication and piezoelectric property of PMN-PT nanofibers. Nano Energy 1, 602–607 (2012).

[b19] Park J. Y., Choi S. W. & Kim S. S. A model for the enhancement of gas sensing properties in SnO_2_-ZnO core–shell nanofibres. J. Phys. D: Appl. Phys. 44, 205403 (2011).

[b20] Tian Z. R. R. *et al.* Complex and oriented ZnO nanostructures. Nat. Mater. 2, 821–826 (2003). 14634640 10.1038/nmat1014

[b21] Choi S. W., Park J. Y. & Kim S. S, Synthesis of SnO_2_-ZnO core–shell nanofibers *via* a novel two-step process and their gas sensing properties. Nanotechnology 20, 465603 (2009). 19847030 10.1088/0957-4484/20/46/465603

[b22] Song X. F., Pan J., Xiao L. S., Gao L. & Mathur S. A hierarchical hybrid design for high performance tin based Li-ion battery anodes. Nanotechnology 24, 205401 (2013). 23598519 10.1088/0957-4484/24/20/205401

[b23] Gong J. Y., Guo S. R., Qian H. S., Xu W. H. & Yu S. H. A general approach for synthesis of a family of functional inorganic nanotubes using highly active carbonaceous nanofibres as templates. J. Mater. Chem. 19, 1037–1042 (2009).

[b24] Jolivet J. P., Gzara M., Mazieres J. & Lefebvre J. Physicochemical Study of Aggregation in Silver Colloids. J. Colloid Interface Sci. 107, 429–441 (1985).

[b25] Hidber P. C., Graule T. J. & Gauckler L. J. Citric Acid–A Dispersant for Aqueous Alumina Suspensions. J. Am. Ceram. Soc. 79, 1857–1867 (1996).

[b26] López-Macipe A., Gómez-Morales J. & Rodríguez-Clemente R., The Role of pH in the Adsorption of Citrate Ions on Hydroxyapatite. J. Colloid Interface Sci. 200, 114–120 (1998).

[b27] Zhou P., Yu J. G. & Jaroniec M., All-Solid-State Z-Scheme Photocatalytic Systems. Adv. Mater. 26, 4920–4935 (2014). 24888530 10.1002/adma.201400288

[b28] Wang X. J., Wan X. L., Xu X. N. & Chen X. N., Facile fabrication of highly efficient AgI/ZnO heterojunction and its application of methylene blue and rhodamine B solutions degradation under natural sunlight. Appl. Surf. Sci. 321, 10–18 (2014).

[b29] Yu J. G., Low J. X., Xiao W., Zhou P. & Jaroniec M. Enhanced Photocatalytic CO2‐Reduction Activity of Anatase TiO2 by Coexposed {001} and {101} Facets. J. Am. Chem. Soc. 136, 8839–8842 (2014). 24918628 10.1021/ja5044787

[b30] 27. Tian W. *et al.* Low-Cost Fully Transparent Ultraviolet Photodetectors Based on Electrospun ZnO-SnO_2_ Heterojunction Nanofibers. Adv. Mater. 25, 4625–4630 (2013). 23836722 10.1002/adma.201301828

[b31] Guo P. H., Jiang J. G., Shen S. H. & Guo L. J. ZnS/ZnO heterojunction as photoelectrode: Type II band alignment towards enhanced photoelectrochemical performance. Int. J. Hydrogen Energy 38, 13097–13103 (2013).

[b32] Ong W. L., Gao M. & Ho G. W. Hybrid organic PVDF–inorganic M–rGO–TiO_2_ (M = Ag, Pt) nanocomposites for multifunctional volatile organic compound sensing and photocatalytic degradation–H_2_ production. Nanoscale 5, 11283–11290 (2013). 24091468 10.1039/c3nr03276k

[b33] Ong W. L., Natarajan S., Kloostra B. & Ho G. W. Metal nanoparticle-loaded hierarchically assembled ZnO nanoflakes for enhanced photocatalytic performance. Nanoscale 5, 5568–5575 (2013). 23681417 10.1039/c3nr00043e

[b34] Herrmann J. M. *et al.* Characterization and photocatalytic activity in aqueous medium of TiO_2_ and Ag-TiO_2_ coatings on quartz. Appl. Catal. B: Environ. 13, 219–228 (1997).

[b35] Ma B. J., Lin K. Y., Su W. G. & Liu W. Y. One-pot synthesis of ZnO/ZnGa2O4 heterojunction with X/XY structure for improved photocatalytic activity. Appl. Surf. Sci. 317, 682–687 (2014).

[b36] Zhu H. Y., Ling X.,. Ru J., Zeng G. M. & Liu L. Efficient decolorization of azo dye solution by visible light-induced photocatalytic process using SnO2/ZnO heterojunction immobilized in chitosan matrix. Chem. Eng. J. 172, 746–753 (2011).

[b37] Wang J., Gao M. M. & Ho G. W., Bidentate-complex-derived TiO_2_/carbon dot photocatalysts: *in situ* synthesis, versatile heterostructures, and enhanced H_2_ evolution. J. Mater. Chem. A 2, 5703–5709 (2014).

[b38] Gao M. M., Peh C. K. N., Ong W. L. & Ho G. W. Green chemistry synthesis of a nanocomposite graphene hydrogel with three-dimensional nano-mesopores for photocatalytic H_2_ production. RSC Adv. 3, 13169–13177 (2013).

[b39] Ahn S. H., Kim D. J., Chi W. S. & Kim J. H. One-Dimensional Hierarchical Nanostructures of TiO_2_ Nanosheets on SnO_2_ Nanotubes for High Efficiency Solid-State Dye-Sensitized Solar Cells. Adv. Mater. 25, 4893–4897 (2013). 23857743 10.1002/adma.201302226

[b40] Moe K. & Ho G. W. Transmission/absorption measurements for *in situ* monitoring of transparent conducting Ga:ZnO films grown *via* aqueous methods. J. Mater. Chem. A 1, 14239–14245 (2013).

[b41] Kushwaha A. & Aslam M. ZnS shielded ZnO nanowire photoanodes for efficient water splitting. Electrochimica Acta 130, 222–231 (2014).

