# Supplementary material for: Hierarchical Assembly of SnO2/ZnO Nanostructures for Enhanced Photocatalytic Performance
Source: Sci Rep. 2015 Jun 25;5:11609. doi: 10.1038/srep11609 (PMC4479871; doi:10.1038/srep11609)
Supplement: Supplementary Information [file srep11609-s1.doc]

**Supplementary Information**

**Hierarchical Assembly of** **SnO_2_/ZnO Nanostructures for Enhanced Photocatalytic Performance**

*Liangliang Zhu^1^, Minghui Hong^1,2^ and Ghim Wei Ho^1,2,3^**

^1^ Department of Electrical and Computer Engineering, National University of Singapore, 4 Engineering Drive 3, 117576, Singapore

^2^ Engineering Science Programme, National University of Singapore, 9 Engineering Drive 1, 117576, Singapore

^3^ Institute of Materials Research and Engineering, A*STAR (Agency for Science, Technology and Research), 3 Research Link, 117602, Singapore

1. **Fabrication of pure SnO_2_ Nanofibers (NFs)**

Pure SnO_2_ NFs were prepared as SZ-10 NFs in the absence of Zn(NO_3_)_2_·6H_2_O by the electrospinning technique. In a typical run, 0.9 g of SnCl_2_·2H_2_O were added into the 5 g of ethanol and 6 g DMF solution under vigorously stirring for 3 h at 60 ^o^C. Subsequently, 1.2 g of PVP was added and the mixture was subsequently magnetic stirred for more 3 h at the same temperature to obtain a homogenous and clear precursor solution. Then, electrospinning was carried out at an applied voltage of 18 kV and a flow rate of 1 mL h^-1^. The distance between needle tip and aluminum foil collector was 15 cm. Finally, the as-spun NFs were calcined in air at 500 ^o^C for 2 h in a furnace, at temperature ramp rate of 5 ^o^C min^-1^ to obtain pure SnO_2_ NFs.

Figure S1 show the typical scanning electron microscopy (SEM) images of pure SnO_2_ NFs before and after annealing. From the Figure S1a, it could be observed that these randomly oriented NFs with the average diameter of *ca.* 100 nm had a smooth and uniform surface due to the amorphous nature of the PVP/SnCl_2_ composite NFs. After annealing, as shown in Figure S1b, the pure SnO_2_ NFs formed and fibrous structure is well preserved with the average diameter exhibited shrinkage to *ca.* 62 nm due to the decomposition of PVP.


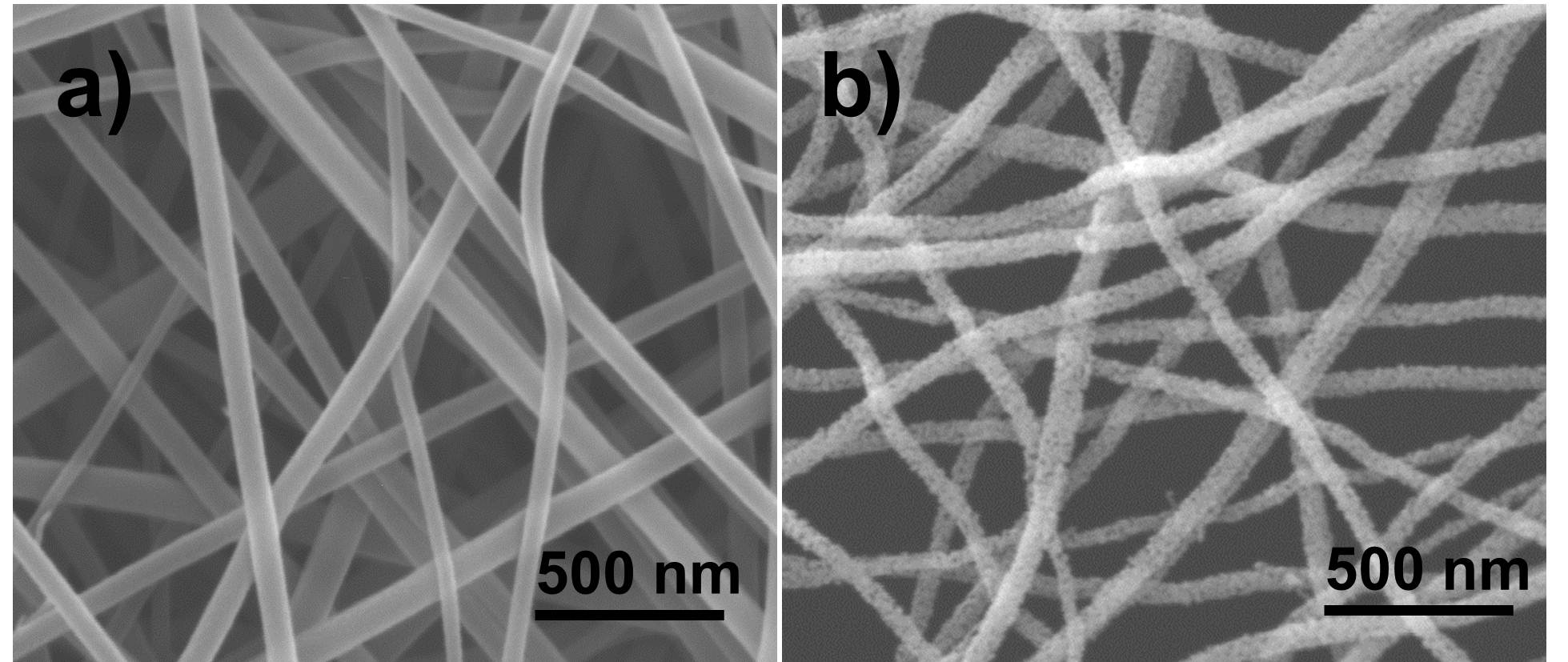


**Figure S1.** The SEM images of pure SnO_2_ NFs before (a) and after (b) anneal.

1. **Synthesis of pure SnO_2_/ZnO nanorods (NRs)**

SnO_2_/ZnO NRs hierarchical nanostructure was synthesized under the same condition of SZ-10/ZnO NRs which is described in experimental section except SZ-10 NFs were replaced by pure SnO_2_ NFs.


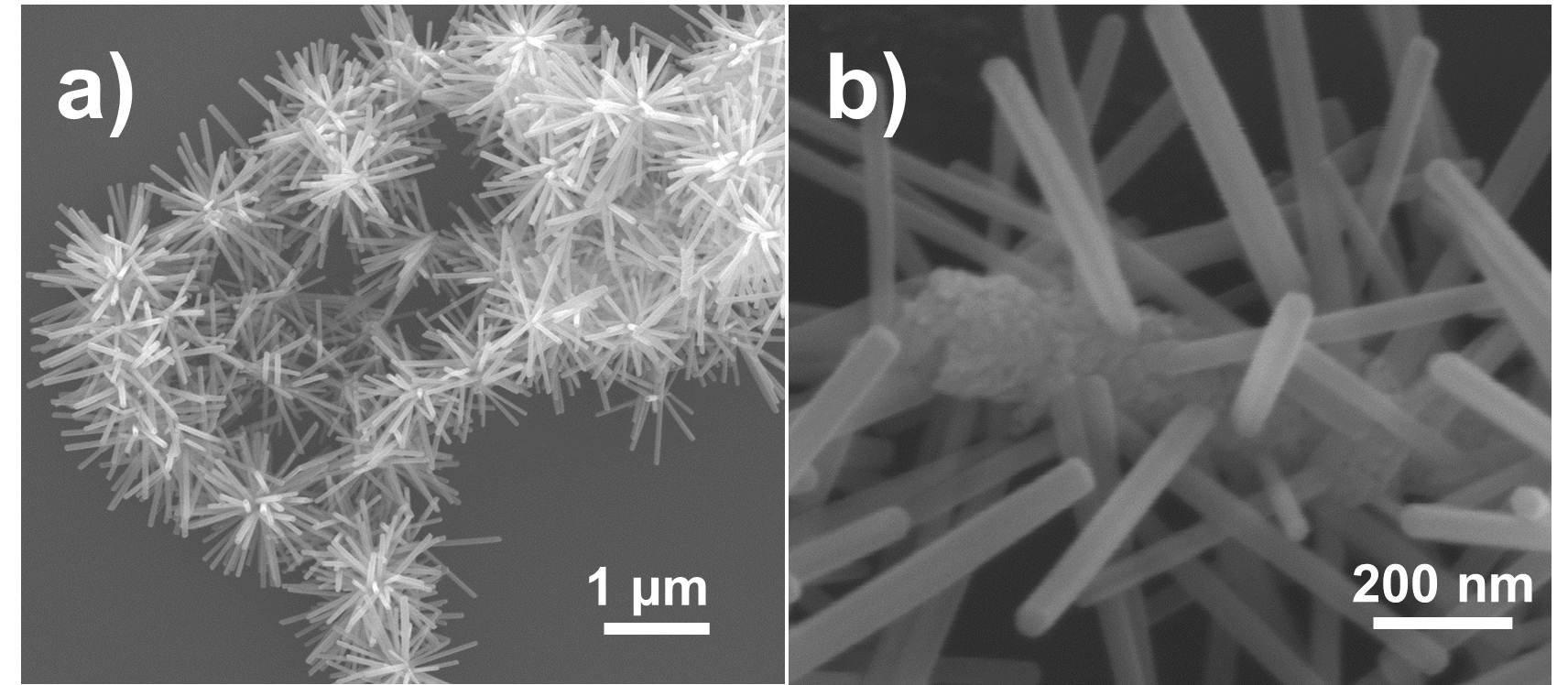


**Figure S2.** The SEM images of SnO_2_/ZnO NRs

1. **TEM image of SnO_2_/ZnO NRs**


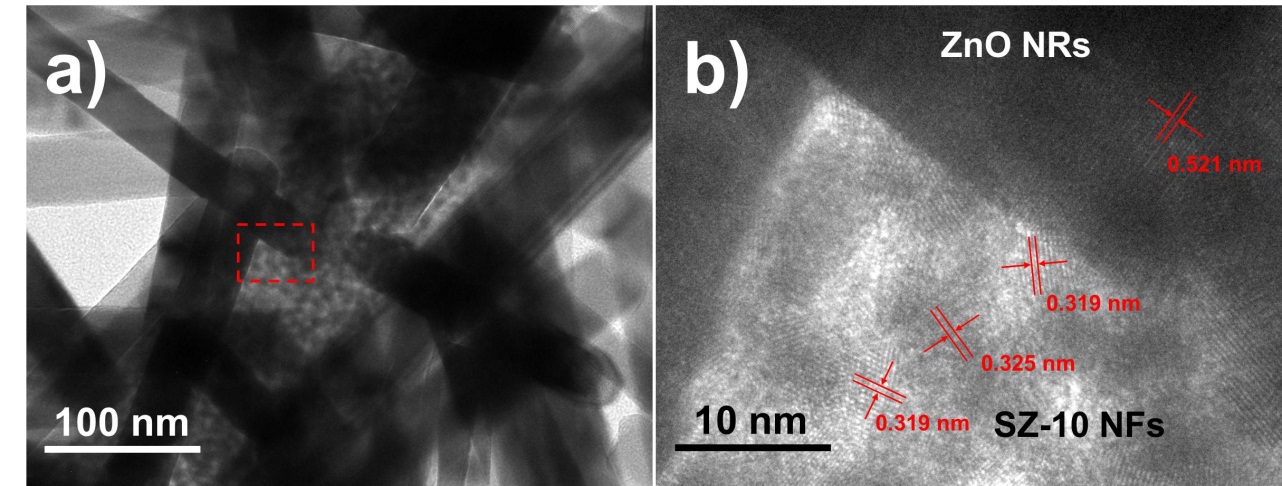


**Figure S3.** (a) The low resolution TEM image of SnO_2_/ZnO NRs. (b) The high resolution TEM image taken from junction of SZ-10 NFs and ZnO NRs.

1. **XRD spectrum of pure SnO_2_ NFs**

Figure S4 depicts the X-Ray photoelectron spectroscopy (XRD) spectrum of the pure SnO_2_ NFs. The diffraction peaks at 26.6, 33.9, 38.1, 51.8, 54.8, 58.0, 61.9, 64.8, 66.1, 71.5 and 78.8^o^ can be indexed to the (110), (101), (200), (211), (220), (002), (310), (112), (301), (202) and (321) crystal planes of the rutile phase SnO_2_ (JCPDS No. 41-1445).


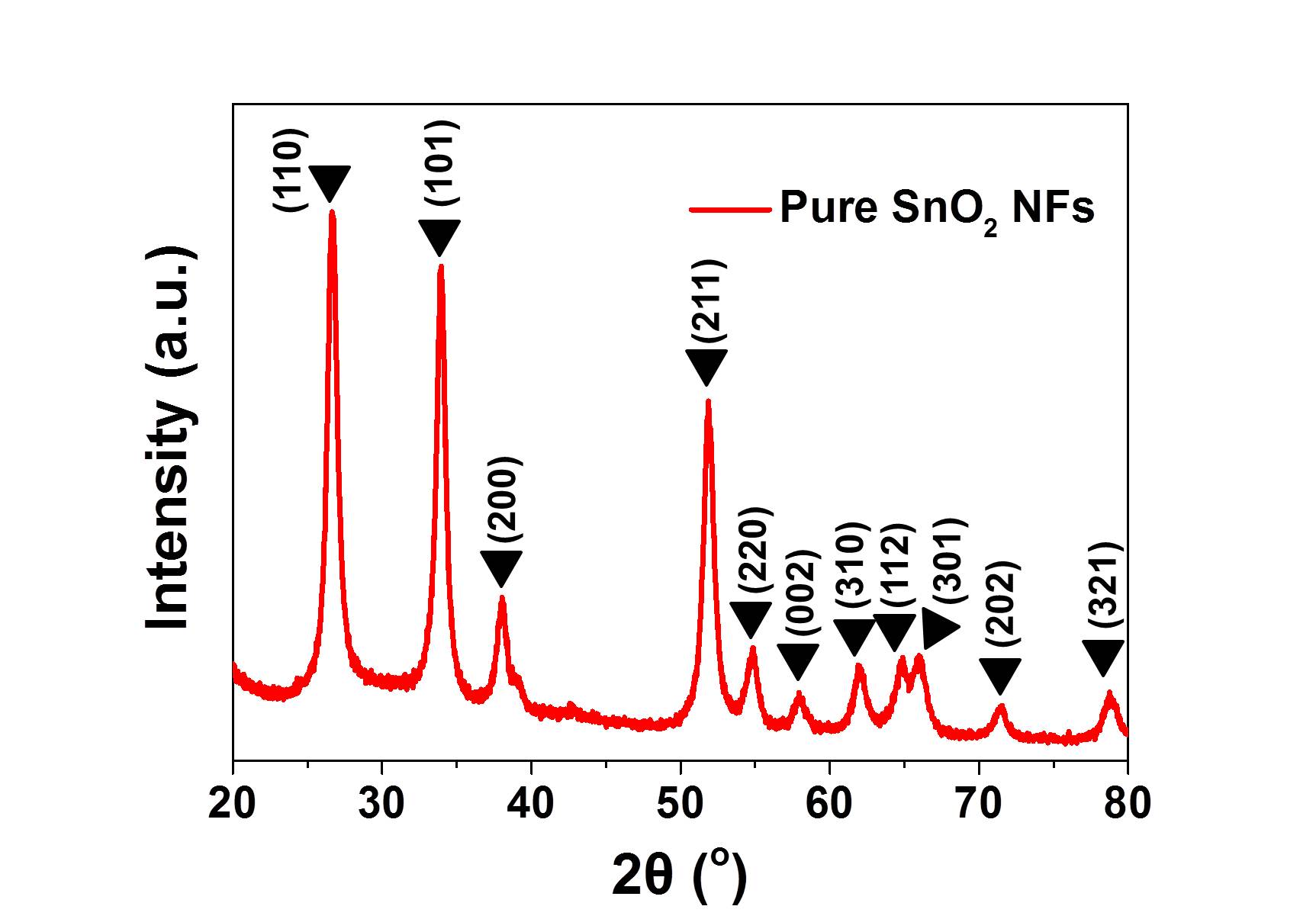


**Figure S4.** The XRD spectrum of pure SnO_2_ NFs.

1. **UV-Vis spectra of SZ-10 NFs, SZ-10/ZnO NRs and SZ-10/ZnO NSs**


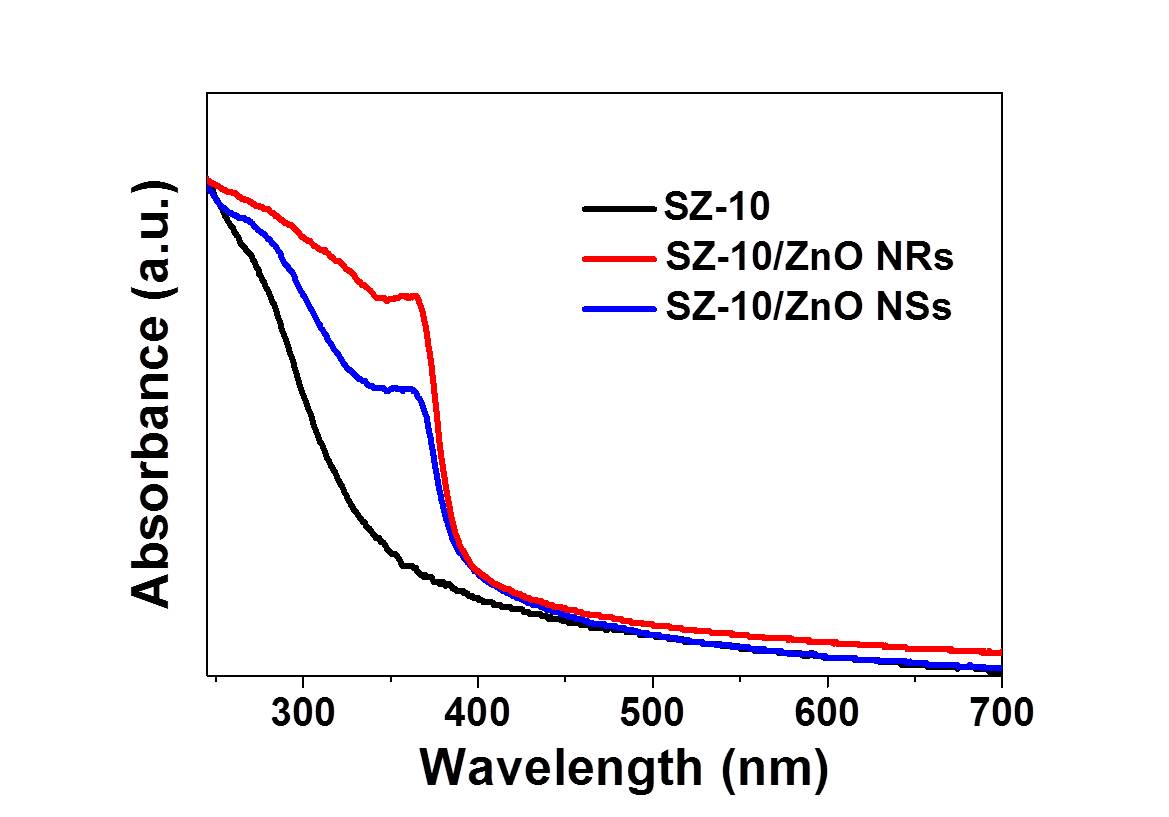


**Figure S5.** The UV-Vis spectra of SZ-10 NFs, SZ-10/ZnO NRs and SZ-10/ZnO NSs.

1. **Synthesis of SZ-10/ZnO NRs**


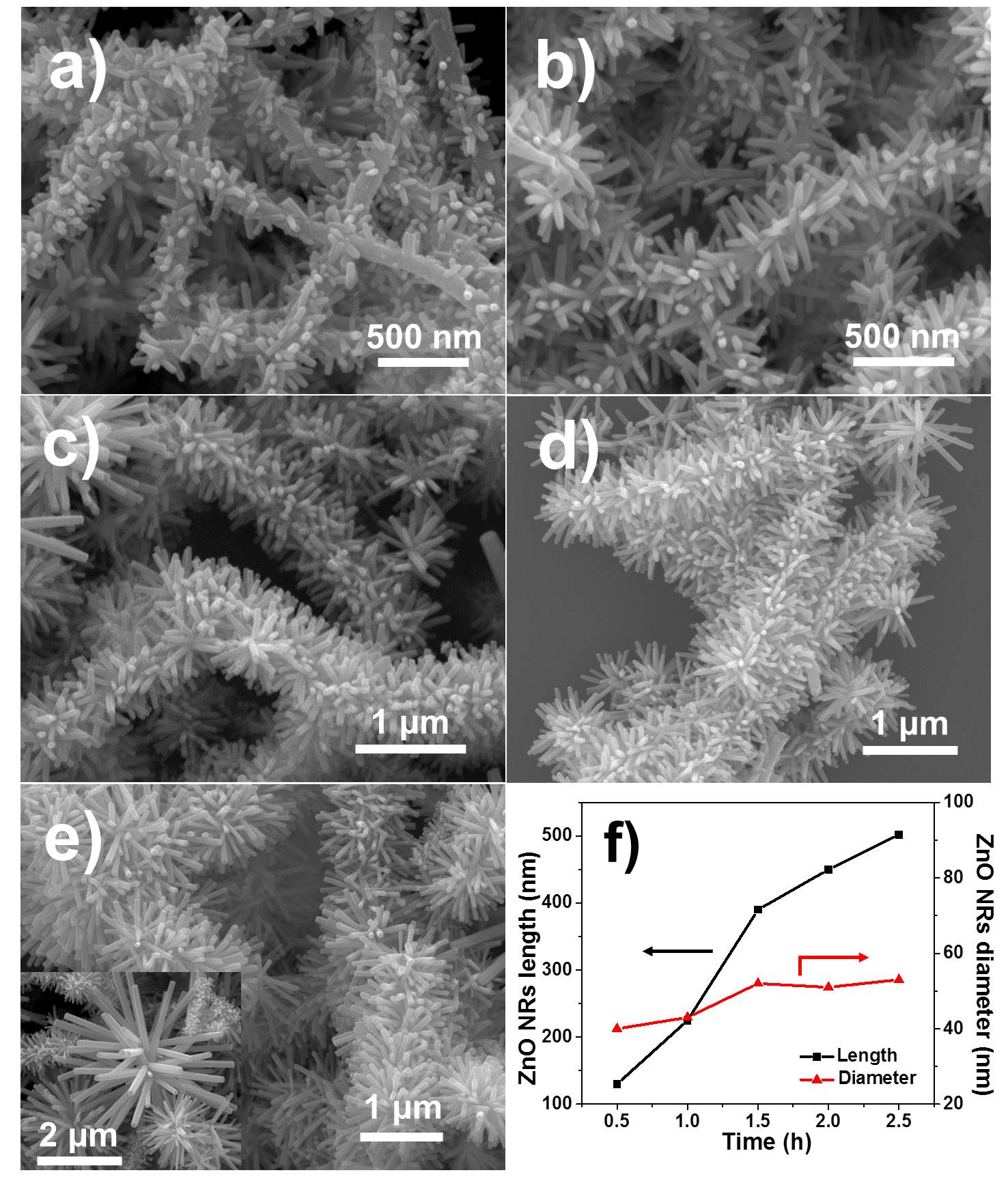


**Figure S6.** The SEM images for SZ-10/ZnO NRs treated by different hydrothermal time: (a) 0.5 h, (b) 1 h, (c) 1.5 h, (d) 2 h and (e) 2.5 h. (f) The length and diameter of the ZnO NRs over the hydrothermal time.

1. **Synthesis of SZ-10/ZnO NSs**


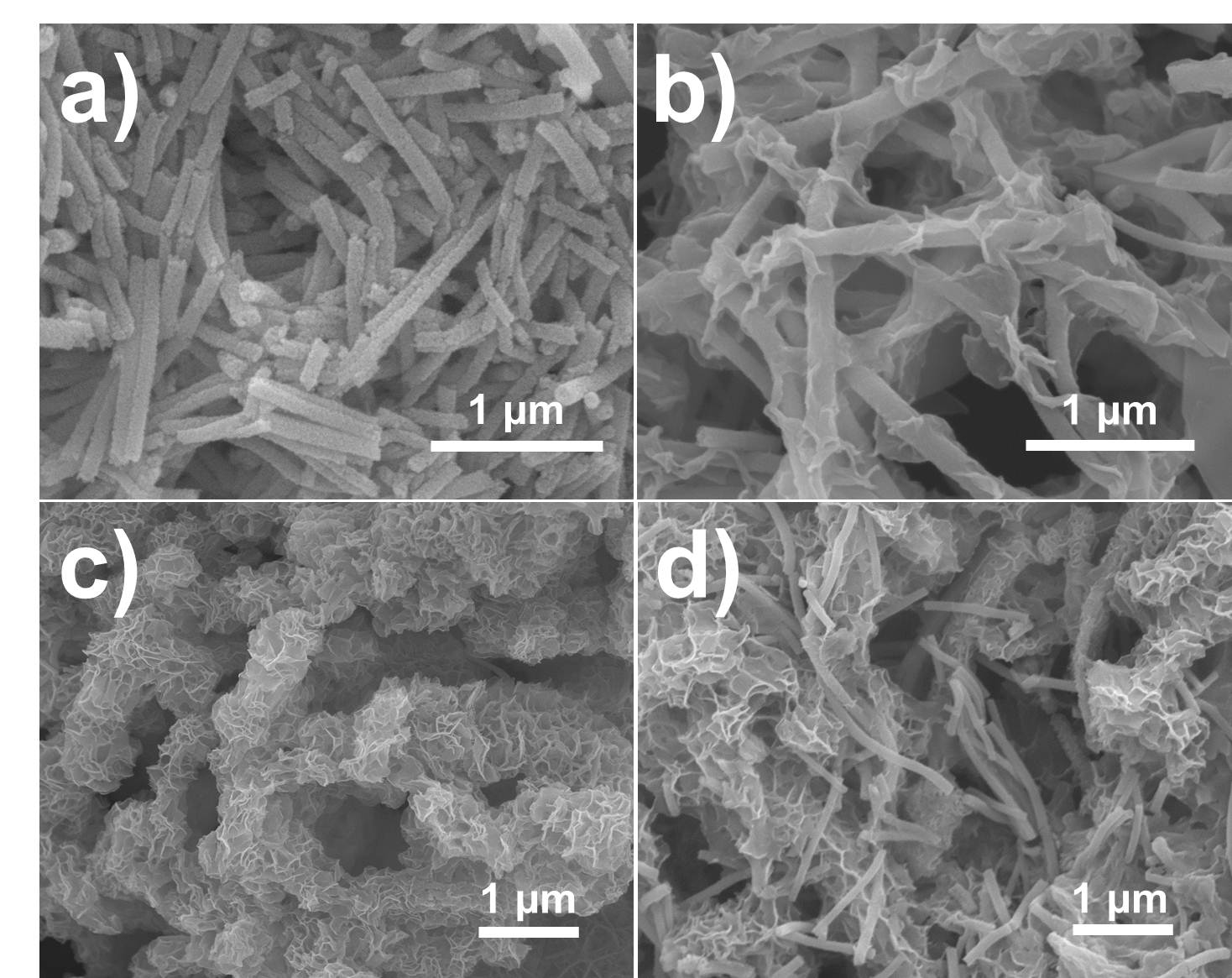


**Figure S7.** The SEM images for SZ-10/ZnO NSs synthesized using the different concentrations of sodium nitrate: (a) 0.3 mol/L, (b) 0.1 mol/L, (c) 0.05 mol/L and (d) 0.025 mol/L.

1. **XPS spectra of SZ-10/ZnO NRs**

To further characterize the product, XPS analysis was conducted to investigate the surface compositions and chemical state of the as-prepared SZ-10/ZnO NRs composite. Figure S8a shows that the Sn 3d spectrum of the sample which appears as a spin-orbit doublet at ~486.8 eV (3d_5/2_) and ~495.3 eV (3d_3/2_) and is in good agreement with the values given in literature.^1^ The binding energy at 1022.2 eV (Figure S8b) is identified as Zn 2p_3/2_, indicating a normal state of Zn^2+^ in the SnO_2_/ZnO hierarchical structures.^2^ The O_1s_ binding energy of 530.7 eV (Figure S8c) indicates that oxygen atoms exist as O_2_^-^ species while the 531.8 eV is attributed to OH^-^ hydroxyl groups or by chemisorbed molecular oxygen (O_2_^-^) in the products.^3^


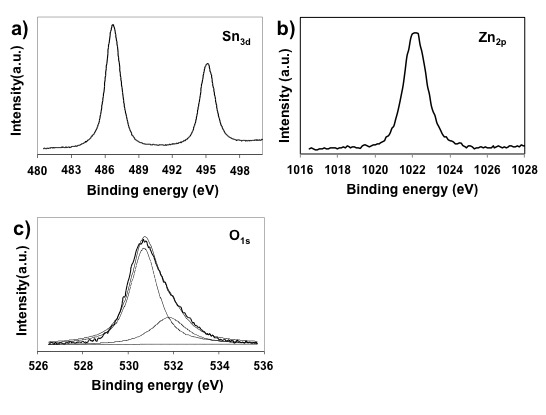


**Figure S8**. XPS spectra of the SnO_2_/ZnO NRs: (a) Sn 3d spectra; (b) Zn 2p spectra; (c) O 1s spectra

1. **Elemental analysis and photocatalytic properties of SZ-10/ZnO composites**

Table S1 Elemental analysis of the SZ-10, SZ-10/ZnO NRs and NSs^a^

| **Element**  (wt%) | **SZ-10 NFs** | **SZ-10/ZnO NRs** | | | |  | **SZ-10/ZnO NSs** | | | |
| --- | --- | --- | --- | --- | --- | --- | --- | --- | --- | --- |
|  |  | **0.5 h** | **1 h** | **1.5 h** | **2 h** |  | **1.5 h** | **3 h** | **4.5 h** | **6 h** |
| **Sn** | 59.54 | 14.09 | 12.28 | 5.47 | 4.33 |  | 20.14 | 5.55 | 3.07 | 3.02 |
| **Zn** | 4.01 | 56.96 | 59.35 | 68.72 | 68.82 |  | 36.08 | 47.36 | 49.82 | 49.97 |
| **O** | 36.54 | 24.82 | 23.86 | 23.08 | 23.68 |  | 33.98 | 31.54 | 35.50 | 33.67 |

^a^The rest ratios belong to C or N elements from the precursors.

Table S1 revealed the changes of weight ratio of Sn, Zn and O elements with the hydrothermal reaction time. The compositions of SZ-10 NFs are Sn 59.54 wt%, Zn 4.01 wt% and O 36.54 wt%, respectively, indicating the incorporation of ZnO into the SnO_2_ NFs. For both SZ-10/ZnO NRs and NSs samples, the content of Zn element increased dramatically at the beginning of the secondary structures growth, 56.96 wt% after 0.5 h and 36.08 wt% after 1.5 h were achieved respectively. Consequently, the Zn content increased slowly and stabilized at *ca.* 69 wt% and 50 wt%, respectively, which may due to the full growth of ZnO NRs.


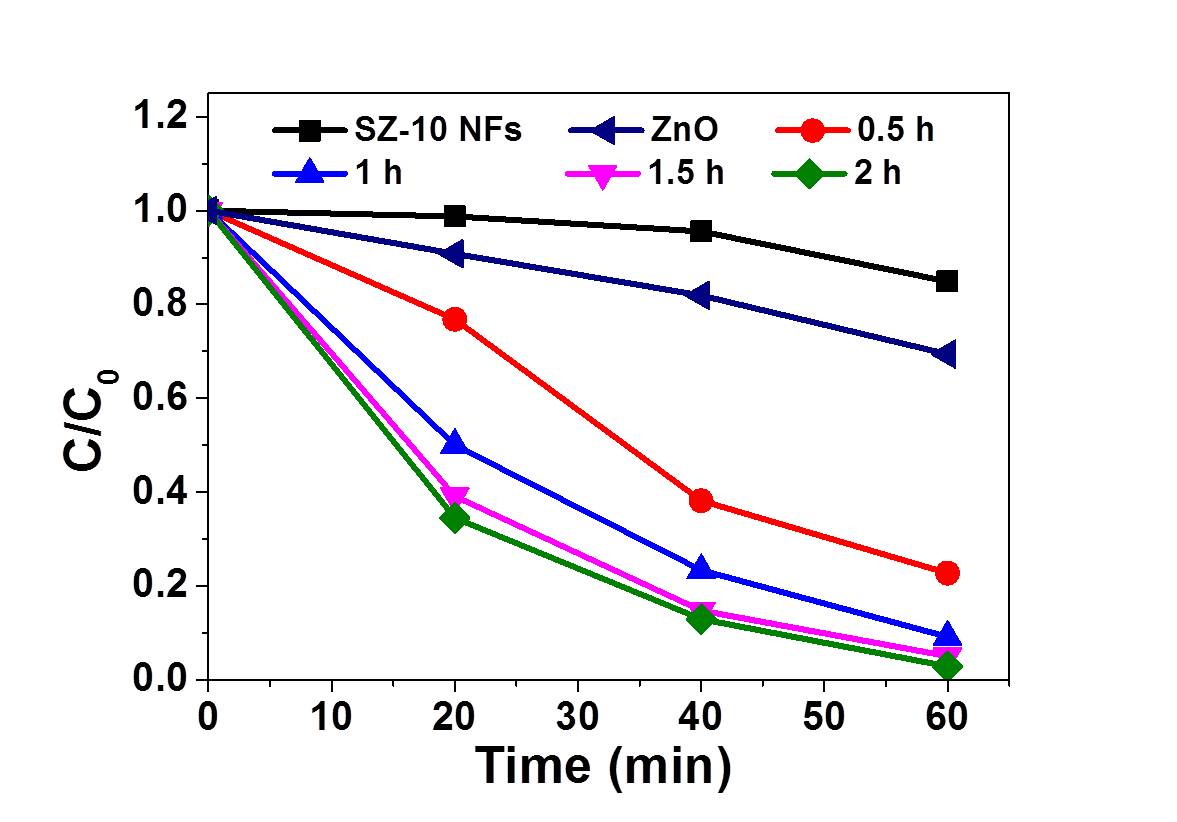


**Figure S9.** Degradation kinetics of time evolution MO photodegradation study in presence of SZ-10/ZnO NRs under different growth time (0.5, 1, 1.5 and 2 h).

The photocatalytic capabilities of the SZ-10/ZnO NRs in the different growth stages (of different SnO_2_/ZnO ratio as reflected in table S1) were also investigated *via* MO dye degradation. The degradation measurements were carried out under the same condition of SZ-10/ZnO NRs (2 h) which is described in experimental section. Figure S9 exhibits the content changes of MO in the presence of the composites. Compared to the pristine SZ-10 NFs, the photocatalytic performances of MO degradation enhanced after the secondary ZnO NRs growth. However, after 1 h hydrothermal treatment, the results of MO degradation is similar (1.5 and 2 h) which due to the full formation of heterojunction interface between SnO_2_ NFs and ZnO NRs, which is consistent with the results of diameter analysis of secondary ZnO NRs presented in Figure S6. The best photocatalytic activity is achieved when the ZnO NRs is fully grown at 5 wt% Sn and 69 wt% Zn composition.

References

(1) Ahn, H.J.; Choi, H.C.; Park, K.W.; Kim, S.B.; Sung, Y.E. Investigation of the Structural and Electrochemical Properties of Size-Controlled SnO2 Nanoparticles. *J. Phys. Chem. B* **108**, 9815-9820 (2004).

(2) Zhang, Z. Y. et al. Electrospun Nanofibers of ZnO-SnO_2_ Heterojunction with High Photocatalytic Activity. *J. Phys. Chem. C* **114**, 7920–7925 (2010).

(3) Balachandran, S., Selvam, K., Babub, B. & Swaminathan, M. The simple hydrothermal synthesis of Ag–ZnO–SnO_2_ nanochain and its multiple applications. *Dalton Trans.* **42**, 16365–16374 (2013).
